# Neutralizing antibody titres in SARS-CoV-2 infections

**DOI:** 10.1038/s41467-020-20247-4

**Published:** 2021-01-04

**Authors:** Eric H. Y. Lau, Owen T. Y. Tsang, David S. C. Hui, Mike Y. W. Kwan, Wai-hung Chan, Susan S. Chiu, Ronald L. W. Ko, Kin H. Chan, Samuel M. S. Cheng, Ranawaka A. P. M. Perera, Benjamin J. Cowling, Leo L. M. Poon, Malik Peiris

**Affiliations:** 1 School of Public Health, The University of Hong Kong, Special Administrative Region of Hong Kong, Hong Kong, China; 2 Infectious Diseases Centre, Princess Margaret Hospital, Hospital Authority of Hong Kong, Special Administrative Region of Hong Kong, Hong Kong, China; 3 Department of Medicine and Therapeutics, Prince of Wales Hospital, Chinese University of Hong Kong, Hong Kong, China; 4 Department of Paediatric and Adolescent Medicine, Princess of Margaret Hospital, Hospital Authority of Hong Kong, Special Administrative Region of Hong Kong, Hong Kong, China; 5 Department of Paediatrics, Queen Elizabeth Hospital, Hospital Authority of Hong Kong, Special Administrative Region of Hong Kong, Hong Kong, China; 6 Department of Paediatric and Adolescent Medicine, The University of Hong Kong and Queen Mary Hospital, Hospital Authority of Hong Kong, Special Administrative Region of Hong Kong, Hong Kong, China; 7 HKU-Pasteur Research Pole, The University of Hong Kong, Special Administrative Region of Hong Kong, Hong Kong, China

**Keywords:** SARS-CoV-2, Medical research

## Abstract

The SARS-CoV-2 pandemic poses the greatest global public health challenge in a century. Neutralizing antibody is a correlate of protection and data on kinetics of virus neutralizing antibody responses are needed. We tested 293 sera from an observational cohort of 195 reverse transcription polymerase chain reaction (RT-PCR) confirmed SARS-CoV-2 infections collected from 0 to 209 days after onset of symptoms. Of 115 sera collected ≥61 days after onset of illness tested using plaque reduction neutralization (PRNT) assays, 99.1% remained seropositive for both 90% (PRNT_90_) and 50% (PRNT_50_) neutralization endpoints. We estimate that it takes at least 372, 416 and 133 days for PRNT_50_ titres to drop to the detection limit of a titre of 1:10 for severe, mild and asymptomatic patients, respectively. At day 90 after onset of symptoms (or initial RT-PCR detection in asymptomatic infections), it took 69, 87 and 31 days for PRNT_50_ antibody titres to decrease by half (T_1/2_) in severe, mild and asymptomatic infections, respectively. Patients with severe disease had higher peak PRNT_90_ and PRNT_50_ antibody titres than patients with mild or asymptomatic infections. Age did not appear to compromise antibody responses, even after accounting for severity. We conclude that SARS-CoV-2 infection elicits robust neutralizing antibody titres in most individuals.

## Abstract (web summary)

Here, the authors perform plaque reduction neutralization (PRNT) assays quantitating SARS-CoV-2 specific neutralizing antibodies from 195 patients in different disease states and find that patients with severe disease exhibit higher peaks of neutralizing antibody titres than patients with mild or asymptomatic infections and that serum neutralizing antibody persists for over 6 months in most people.

## Introduction

Virus neutralizing antibody is likely to be a key correlate of protection for COVID-19^1^ and data on kinetics of virus neutralizing antibody responses are needed^2^. Development of population immunity, achieved through natural infection, or preferably through vaccination is essential for combating the COVID-19 pandemic. Antibody and T cell responses are the main arms of the adaptive immune response. The mechanisms of protective immunity and the duration of such protection against COVID-19 remain to be elucidated. In other respiratory infections such as influenza, antibody responses are known to protect against infection and T cell responses modulate the severity of disease^3^. Correlates of protection provided by antibody are well established for influenza, and approximately 50% of those with serum haemagglutination inhibition antibody titers of 1:40 were protected from infection, the proportion protected increasing progressively with higher antibody titers^4–6^. Patients with SARS developed both neutralizing antibody as well as T cell immunity, with neutralizing antibody and T cell responses being detectable for around 3 years and >17 years post-infection, respectively^7,8^. Since SARS-CoV-1 is no longer circulating in humans, there is no direct evidence of protection from reinfection, if any, provided by antibody or T cell responses. In experimental animal models, the spike protein was necessary and sufficient to elicit neutralizing antibody and protection from challenge with SARS-CoV-1. Other virus structural proteins N, M, and E were not able to mediate protection in the absence of S protein^9^.

In experimental animal models of SARS-CoV-2 infection in Golden Syrian hamsters and in non-human primates, prior infection as well as transfer of immune serum protected animals from disease though it did not provide sterilizing immunity^10,11^. Rhesus macaques immunized with SARS-CoV-2 spike were protected from challenge and protection correlated with neutralizing antibody levels^12^. Evidence of protection against reinfection of humans with SARS-CoV-2 is limited. Pre-existing SARS-CoV-2 neutralizing antibody was protective from reinfection in an outbreak of SARS-CoV-2 on a fishing vessel^1^. It is noted however, that the association of protection with neutralizing antibody may not necessarily be causal^13^ as other forms of immunity (e.g., T cell immunity, non-neutralizing antibody) may contribute to protection. Neutralizing monoclonal antibodies protect rhesus macaques and mice from disease after experimental challenge^14^. The therapeutic use of convalescent plasma has been given Emergency Use Authorization by the U.S. Food and Drug Administration^15^. Taken together, these observations suggest that neutralizing antibody is a key correlate of protection against SARS-CoV-2 infection, although it may not be the only correlate of protection against SARS-CoV-2.

Many studies have reported antibody responses in COVID-19 patients using ELISA or other binding assays, but there are fewer reports using virus neutralization tests. Patients with COVID-19 infection develop detectable SARS-CoV-2 neutralizing antibody responses with some having detectable antibody at the end of the 1st week of illness, and almost all having neutralizing antibody after 4 weeks of illness^16–21^. The magnitude of the antibody responses and the proportion of patients developing antibody responses have varied. Severely ill patients are reported to have higher peak neutralizing antibody titers^17,19,20^. The methods used for detecting neutralizing antibody has also varied. These include the use of pseudoparticle neutralization (ppNT), microneutralization, fluorescent focus reduction assays, microneutralization assays, and plaque reduction neutralization tests (PRNT). Pseudoparticle neutralization tests are convenient and do not require bio-safety level 3 containment, but it is not clear how closely different types of virus pseudoparticles expressing SARS-CoV-2 spike protein mimics authentic virus, or how results from one pseudoparticle assay compares with another. Even with neutralization of live virus, microneutralization tests were found to be less sensitive than plaque reduction neutralization assays, which are regarded as the “gold-standard” for neutralizing antibody testing^16^. The major outstanding question is the duration of these neutralizing antibody responses. There are reports of rapid waning of antibody with some reports claiming that a third of patients have lost pseudoparticle neutralizing antibody by around 1–2 months after onset of illness^22^. If true, such findings have major implications for the duration of protective immunity from reinfection, and the likely success of vaccination in prevention from reinfection and disease. It is therefore essential that the duration of neutralizing antibody responses are assessed using live virus neutralization assays.

In this work, we used 293 sera from 195 individuals with RT-PCR confirmed SARS-CoV-2 infection, 70 of them providing multiple sequential sera, to assess the kinetics of PRNT_50_ and PRNT_90_ antibody titers. The spike protein receptor binding domain (RBD) is the most immune-dominant neutralizing epitope eliciting virus neutralization^23^. Therefore, for comparison, we have also analysed the duration of positivity of ELISA antibody responses to the virus spike RBD in a subset of 231 sera from 150 individuals. We show that SARS-CoV-2 infection elicits robust neutralizing antibody titers in most individuals that last beyond 6 months.

## Results

The RT-PCR confirmed SARS-CoV-infected patients ranged in age from 3 months to 80 years, 118 being males and 77 female (Supplementary Table 1). Thirteen patients had severe illness requiring >3 l of supplemental oxygen/minute, 151 were mild-moderate in severity (symptomatic but not requiring >3 l of supplemental oxygen per minute) and 31 were asymptomatic throughout the infection. Clotted blood was collected for serology. As expected, most of the severely ill patients were aged ≥60 years of age (Supplementary Table 2).

**Fig. 1:**
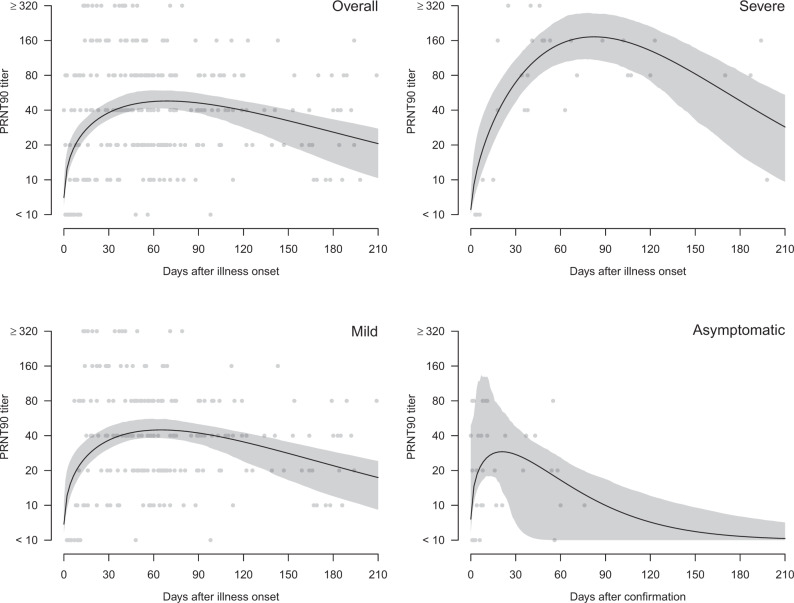
Antibody responses (PRNT_90_) in COVID-19 patients by days after illness onset and severity, Hong Kong (*n* = 293 samples). The black lines showed the fitted values and gray areas showed the 95% confidence intervals. Neutralization tests were carried out in duplicate.

**Fig. 2:**
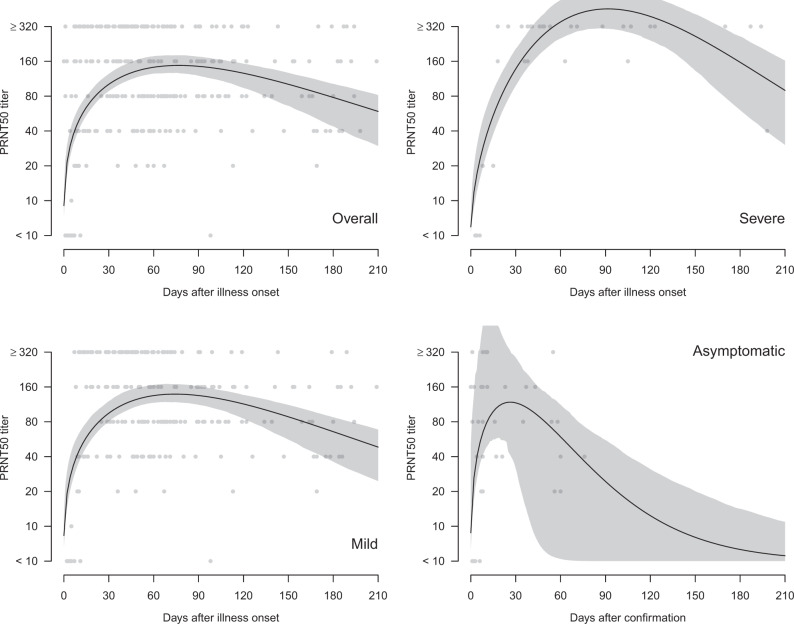
Antibody responses (PRNT_50_) in COVID-19 patients by days after illness onset and severity, Hong Kong (*n* = 293 samples). The black lines showed the fitted values and gray areas showed the 95% confidence intervals. Neutralization tests were carried out in duplicate.

Two hundred and ninety three sera from 195 patients were tested in PRNT assays and the highest serum dilution reducing plaque numbers by 90% (PRNT_90_) and 50% (PRNT_50_) were determined. Of 234 sera collected from individuals 15–209 days after onset of illness, 98.7% and 99.6% were positive by PRNT_90_ and PRNT_50_ antibody tests, respectively (Supplementary Table 3). Of sera collected ≥61 days after onset of illness, 99.1% were positive in PRNT_90_ and PRNT_50_ antibody tests. The individual data-points for PRNT_90_ (Fig. 1) and PRNT_50_ (Fig. 2) for each serum are shown for the cohort as a whole, and stratified into those with severe disease, mild disease, or asymptomatic infections. Patients with severe disease had higher peak PRNT_90_ and PRNT_50_ antibody titers but took longer (around day 90) to reach peak antibody titers than the mild (around day 60) and asymptomatic (around day 30) infections. To obtain a conservative estimate of the duration of antibody responses, we fitted a Weibull curve to the existing data, and we estimate that it takes 292, 295, and 90 days for PRNT_90_ titers to drop to the detection limit of 1:10 in severe, mild and asymptomatic patients respectively. Similarly, it takes 372, 416, and 133 days for PRNT_50_ titers to drop to the detection limit of a titre of 1:10 for severe, mild and asymptomatic patients, respectively. At day 90 after onset of symptoms (or initial detection in asymptomatic infections), PRNT_50_ antibody titers declined by half (*T*_1/2_) by 69, 87, and 31 days in severe, mild, and asymptomatic infections was, respectively. Corresponding *T*_1/2_ for PRNT_90_ antibody for severe and mild infections was 58 and 95 days, respectively, the *T*_1/2_ for asymptomatic infections beyond day 90 being too low to quantitate.

**Fig. 3:**
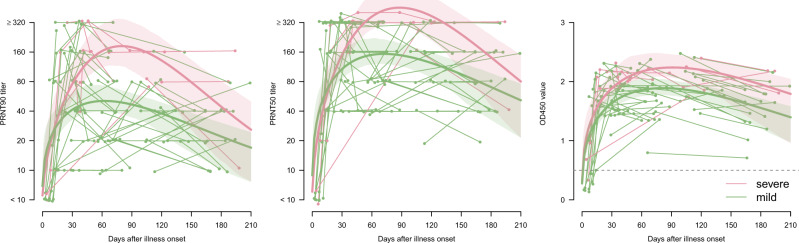
Longitudinal antibody responses (PRNT_90_, PRNT_50_, and ELISA) in COVID-19 patients by days after illness onset and severity, Hong Kong. Data from 61 patients with multiple samples for PRNT_90_ and PRNT_50_ antibody titers and 54 patients with multiple samples for ELISA. Small random noises were added to the PRNT_90_ and PRNT_50_ titers for better presentation. The thick lines showed the fitted values and shaded areas showed the 95% confidence intervals. Neutralization and ELISA assays were carried out in duplicate.

Using a generalized additive mixed model which accounted for longitudinal changes in antibody titers and corticosteroid use, mild cases aged >60 years had mean antibody responses 0.40 (95% CI −0.19 to 0.99) and 0.38 (95% CI −0.16 to 0.92) log2 higher for PRNT_90_ and PRNT_50_ titers respectively, and 0.34 (95% CI 0.17–0.51) higher for ELISA OD values, compared to younger age groups. Among the severe cases, the differences were not statistically significant, being −1.01 (95% CI −2.32 to 0.30) and −0.38 (95% CI −1.23 to 0.47) log2 difference for PRNT_90_ and PRNT_50_ titers, respectively, and 0.06 (95% CI −0.24 to 0.36) difference for ELISA OD values, compared to younger age groups. Most patients with severe disease were treated with corticosteroids. There was apparently no significant independent effect of corticosteroid use on peak antibody titers, although statistical power was suboptimal. We had serial longitudinal sera available from a subset of 70 patients. The numbers of longitudinal sera from the asymptomatic infections were too limited for accurate curve fitting. The longitudinal data from 61 patients (149 sera) with severe and mild disease are shown in Fig. 3 with a Weibull curve fitted to the data. As with the aggregate data, we see that patients with severe disease had higher peak PRNT_90_ and PRNT_50_ titers, peaking later than mild infections. From the fitted curve, we estimate that it takes 282 and 297 days for PRNT_90_ titers to drop to 1:10 for severe and mild patients, respectively. Similarly, it takes 365 and 435 days for PRNT_50_ titers to drop to 1:10 for severe and mild patients.

**Fig. 4:**
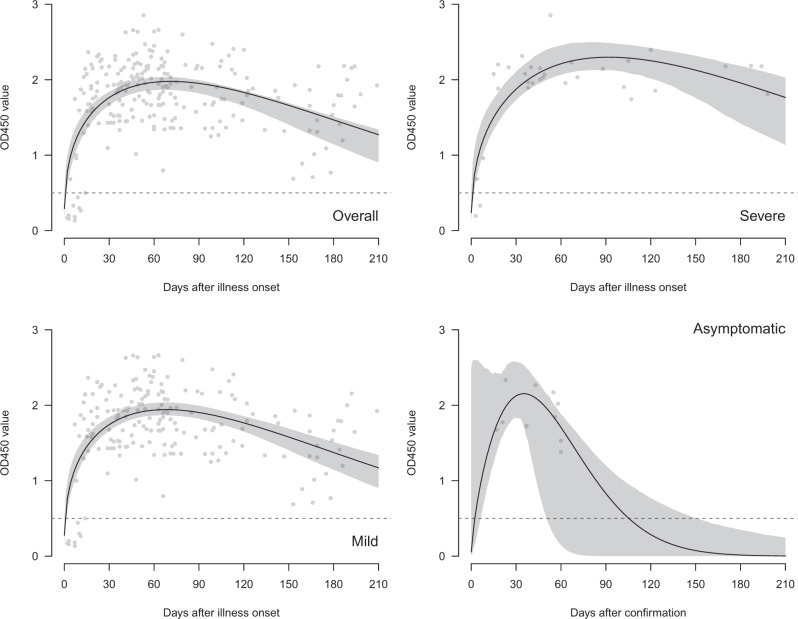
Antibody responses (ELISA) in COVID-19 patients by days after illness onset and severity, Hong Kong (*n* = 231 samples). The black lines showed the fitted values and gray areas showed the 95% confidence intervals. ELISA assays were carried out in duplicate.

Data on spike RBD IgG ELISA were available on 231 sera collected from 150 patients (Fig. 4). Of 209 sera collected from day 15 to 209 after onset of illness, all were positive in the RBD ELISA assay (Supplementary Table 3). Individual ELISA optical density (OD) readings are not quantitative in a manner comparable with PRNT titers, with loss of linearity when OD readings go above 2.0. Extrapolation of the Weibull curve fitted to the aggregate data of severe, mild, and asymptomatic individuals suggests that it takes 448, 337, and 105 days, respectively, for ELISA OD_450_ values to drop to the negative cut-off value (Fig. 4). Longitudinal data from 134 sera from 54 patients with severe or mild disease are shown (Fig. 3) and it is estimated that the RBD ELISA will be negative by approximately 495 and 416 days in severe and mild patients, respectively. The number of longitudinal sera from the asymptomatic individuals were too few to give reliable estimates.

The correlation of PRNT_90_ and PRNT_50_ antibody titers with ELISA OD_450_ is shown in (Supplementary Fig. 1). There is saturation of OD_450_ readings once the OD goes above 2.0, leading to a loss of linearity at higher antibody titers.

## Discussion

The kinetics and duration of neutralizing antibody responses of individuals with SARS-CoV-2 infection is required for understanding a key correlate of protection from reinfection and disease. Plaque reduction neutralization tests are the “gold-standard” for assessing neutralizing antibody titers with 50% reduction of plaque numbers (PRNT_50_) being an established end-point for assessing the serum neutralizing titre. We have determined both the 50% (PRNT_50_) as well as the more stringent 90% (PRNT_90_) plaque reduction endpoints to assess neutralizing antibody titers. We found that almost all individuals in our cohort sampled from day 15 to day 209 days after onset of illness had detectable PRNT_90_ and PRNT_50_ antibody. Importantly, 99.1% of 115 sera collected ≥61 days after onset of illness remained antibody positive in both PRNT_90_ and PRNT_50_ assays. We fitted a Weibull curve to the data to allow us to compare the antibody peak and waning kinetics of those with severe, mild, and asymptomatic infections. As previously reported, patients with severe illness had higher peak titers of PRNT_90_ and PRNT_50_ antibody than did those with mild illness or those with asymptomatic infection^17,19,20^. It is noteworthy that individuals with asymptomatic infection also developed PRNT antibody titers apparently comparable with those with mild illness, but the small numbers of sera tested led to wider confidence bounds. To test the hypothesis that older adults may have lower antibody responses, we investigated the effect of older age on antibody responses, adjusting for disease severity and duration of illness. To our surprise, we found that those aged >60 years had significantly higher mean ELISA antibody responses compared to younger age groups with this difference not reaching statistical significance for the PRNT_90_ and PRNT_50_ antibody titers. Thus, it does not appear that age compromises antibody immune responses to natural infection.

In typical antibody responses, titers peak at around 30–40 days after onset of illness followed by a biexponential waning of antibody titers. There is a period of rapid waning associated with the contraction by apoptosis of the initial pool of antigen specific B cells as the infection has subsided. This is followed by a slower wane of antibody titers, thereafter with the antibody levels being maintained by antigen specific long-lived plasma cells in the bone marrow^24,25^. Our data do not allow us to capture this second phase of the slower antibody waning kinetics for which we need more data in later time periods (beyond day 200) after onset of disease. Therefore our estimates of antibody waning, influenced heavily by the early phase rapid of waning, are likely to be overly conservative.

Antibody reactive to the receptor binding domain of SARS-CoV-1 remained detectable for at least 3 years follow-up with 95% of the convalescent patients being seropositive at 3 years of post-infection^7,26^. The data on neutralizing antibody responses for MERS-CoV has similarities and differences with what is observed here for SARS-CoV-2. While most patients with severe MERS developed strong antibody responses that were long-lasted, a proportion of those with mild infection had very weak or no antibody responses^27,28^. Seasonal human coronaviruses 229E, OC43, HKU1, and NL63 generally cause mild upper respiratory disease and reinfections with the same are common, with median of approximately 30 months between them, although reinfection can occur at intervals as short as 6 months apart associated with rapid antibody waning^29^. Experimental challenge of human volunteers with 229E has demonstrated antibody waning but detectable antibody at the end of one year although such antibody did not prevent experimentally rechallenged volunteers from developing symptomatic reinfection^30^.

Limitations of our study are that we have few sera beyond 200 days after onset of symptoms to define the late antibody waning kinetics. We also have relatively fewer numbers of asymptomatic infections (*n* = 31) compared to those with mild disease (*n* = 151). Thus, our findings on asymptomatic infections need to be interpreted with caution. Since the focus of our study was the kinetics of neutralizing antibody, we did not carry out titrations to define the end-points in our spike RBD ELISA IgG antibody assays; our data being OD values at a single serum dilution (1/100).

In summary, our findings of neutralizing antibody responses in SARS-CoV-2 infections are comparable to what is observed with SARS and other some other viral infections. It is likely that neutralizing antibody will be maintained over the first year after mild or severe disease with higher antibody titers and longer duration of detectable antibody in those with severe disease. It is important to note that even once neutralizing antibody levels have dropped below the detectable threshold, that immune memory will lead to rapid anamnestic antibody responses following re-exposure to the virus, and these are likely to be protective against severe disease. It is also noted that antibody may not confer sterilizing immunity but may prevent from reinfection leading to severe disease. A recent case of asymptomatic SARS-CoV-2 reinfection in a person, who failed to develop neutralizing antibody following his first infection is relevant in this regard^31,32^. Investigation of further cases of reinfection in regard to immune responses and onward transmission will be revealing.

## Methods

### Patients

An observational study was carried out on a cohort of one hundred and ninety five individuals with symptomatic or asymptomatic RT-PCR confirmed SARS-CoV-2 infections from the Princess Margaret, Prince of Wales, Queen Elizabeth and Queen Mary hospitals of the Hospital Authority of Hong Kong consented to participate in this study. Patient recruitment commenced on 21st January 2020 and continued until 31st July 2020. Recruited patients were followed up to 22th September 2020. Written informed consent was obtained and the studies were approved by the institutional review boards of the respective hospitals, viz. Kowloon West Cluster (KW/EX-20-039 (144-27)), Kowloon Central/Kowloon East cluster (KC/KE-20-0154/ER2) and HKU/HA Hong Kong West Cluster (UW 20-273). Clinical management of adult and pediatric patients was decided by the attending clinicians based on the standard of care as recommended by the Central Committee on Infectious Diseases and Emergency Response (CCIDER) of the Hospital Authority of Hong Kong, as revised periodically since February 2020. The antivirals used were interferon, lopinavir/ritonavir (Kaletra) or ribavirin, used individually or in combination.

### Plaque reduction neutralization test (PRNT)

Vero E6 cells (ATCC CRL-1586) were maintained in Dulbecco’s Modified Eagle Medium (DMEM) medium supplemented with 10% fetal bovine serum (FBS) and 100 U/ml of penicillin–streptomycin. The assay was performed in duplicate using 24-well tissue culture plates (TPP Techno Plastic Products AG, Trasadingen, Switzerland) in a biosafety level 3 facility. Serial dilutions of each serum sample was incubated with 30–40 plaque-forming units of virus for 1 h at 37 °C. The virus-serum mixtures were added onto pre-formed Vero E6 cell monolayers and incubated for 1 h at 37 °C in 5% CO_2_ incubator. The cell monolayer was then overlaid with 1% agarose in cell culture medium and incubated for 3 days, at which time the plates were fixed and stained. Antibody titers were defined as the highest serum dilution that resulted in ≥90% (PRNT_90_) reduction or >50% (PRNT_50_) in the number of virus plaques. This method has been extensively validated on SARS-CoV-2 infected and control sera previously^16^.

### SARS-CoV-2 spike receptor binding domain ELISA

Ninety-six-well ELISA plates (Nunc MaxiSorp, Thermo Fisher Scientific) were coated overnight with 100 ng per well of the purified recombinant RBD protein in PBS buffer. The plates were then blocked with 100 μl of Chonblock blocking/sample dilution ELISA buffer (Chondrex Inc, Redmon, US) and incubated at room temperature for 2 h. Each serum or plasma sample was tested in duplicate at a dilution of 1:100 in Chonblock blocking/sample dilution ELISA buffer and 100 µl was added to the wells of each plate for 2 h incubation at 37 °C. After extensive washing with PBS containing 0.1% Tween 20, horseradish peroxidase (HRP)-conjugated goat anti-human IgG (1:5000, GE Healthcare) was added for 1 h at 37 °C. The ELISA plates were then washed five times with PBS containing 0.1% Tween 20. Subsequently, 100 μl of HRP substrate (Ncm TMB One; New Cell and Molecular Biotech Co. Ltd, Suzhou, China) was added into each well. After 15 min incubation, the reaction was stopped by adding 50 μl of 2 M H_2_SO_4_ solution and analysed on a Sunrise (Tecan, Männedorf, Switzerland) absorbance microplate reader at 450 nm wavelength. Normalized results were obtained by calculating the difference between the OD of the purified recombinant protein-coated well and the PBS-coated well. The method was previously validated and reported^16^.

### Statistical analysis

We presented PRNT_90_, PRNT_50_ log titers, and ELISA OD values by severe, mild, and asymptomatic SARS-CoV-2 infections. Nonlinear mixed effects model with Weibull curves were fitted to the data, to model the initial increase, and decay over time and accounted for the correlation among measurements from the same patients. The 95% confidence intervals were constructed using parametric bootstrap based on the estimated values and variance–covariance matrix of the parameters, with 1000 resamples. Based on the fitted Weibull curve, we extrapolated to the time when PRNT titers reach 1:10 and ELISA reaches the negative cut-off threshold. We also presented a subset of the COVID-19 cases where longitudinal data was available. We tested the potential differences in mean antibody response by age for severe and mild cases respectively, using generalized additive mixed effects model accounting for temporal change in antibody response, and use of corticosteroids, using cubic spline function for the antibody response. We also calculated the spearman correlation between PRNT_90_/PRNT_50_ titers and ELISA OD values in those sera when paired data were available.

### Reporting summary

Further information on research design is available in the Nature Research Reporting Summary linked to this article.

## Supplementary information

Supplementary Information

Peer Review File

Reporting Summary

## Data Availability

The source data (individual anonymized patient and linked laboratory data) are provided as a Source Data file available on line. Source data are provided with this paper.

## Source data

Source Data
