# Supplementary material for: Neutralizing antibody titres in SARS-CoV-2 infections
Source: Nat Commun. 2021 Jan 4;12:63. doi: 10.1038/s41467-020-20247-4 (PMC7782739; doi:10.1038/s41467-020-20247-4)

SUPPLEMENTARY INFORMATION

Neutralizing antibody titres in SARS-CoV-2 infections

Eric HY Lau[1], Owen TY Tsang,[2] David SC Hui,[3] Mike YW Kwan,[4] Wai-hung Chan,[5] Susan S Chiu,[6] Ronald LW Ko[1], Kin H Chan[1], Samuel M.S. Cheng[1], Ranawaka APM Perera[1], Benjamin J Cowling[1], Leo LM Poon[1], Malik Peiris[1,7,*].

1. School of Public Health, The University of Hong Kong, Special Administrative Region of Hong Kong, China

2. Infectious Diseases Centre, Princess Margaret Hospital, Hospital Authority of Hong Kong, Special Administrative Region of Hong Kong, China.

3. Department of Medicine and Therapeutics, Prince of Wales Hospital, Chinese University of Hong Kong,

4. Department of Paediatric and Adolescent Medicine, Princess Margaret Hospital, Hospital Authority of Hong Kong, Special Administrative Region of Hong Kong, China.

5. Department of Paediatrics, Queen Elizabeth Hospital, Hospital Authority of Hong Kong, Special Administrative Region of Hong Kong, China.

6. Department of Paediatric and Adolescent Medicine, The University of Hong Kong and Queen Mary Hospital, Hospital Authority of Hong Kong, Special Administrative Region of Hong Kong, China.

7. HKU-Pasteur Research Pole, The University of Hong Kong, Special Administrative Region of Hong Kong, China.

***Address correspondence** to Dr. Malik Peiris, School of Public Health, The University of Hong Kong, Pokfulam, Hong Kong Special Administrative Region. E mail: malik@hku.hk

Key words: COVID-19, SARS-CoV-2, coronavirus, neutralizing antibody, kinetics, protection, immunity.

Supplementary Table 1. Characteristics of patients tested by plaque reduction neutralization tests (PRNT) and spike receptor binding domain ELISA.

	PRNT				ELISA			
	All patients (n=195)		Patients with multiple samples for $PRNT_{50}$ and $PRNT_{90}$ (n=70)		All patients (n=150)		Patients with multiple samples for ELISA (n=55)	
	N	(%)	N	(%)	N	(%)	N	(%)
Age (y)								
≤15	42	(22%)	9	(13%)	6	(4%)	0	(0%)
16-60	119	(61%)	44	(63%)	110	(73%)	39	(71%)
>60	34	(17%)	17	(24%)	34	(23%)	16	(29%)
Male	118	(61%)	38	(54%)	92	(61%)	29	(53%)
With underlying conditions	49/194	(25%)	16	(23%)	45	(30%)	15	(27%)
Antiviral treatment	100	(51%)	42	(60%)	92	(61%)	38	(69%)
Corticosteroid treatment	12	(6%)	7	(10%)	12	(8%)	7	(13%)
Worst condition								
Severe	13	(7%)	9	(13%)	13	(9%)	9	(16%)
Mild	151	(77%)	52	(74%)	128	(85%)	45	(82%)
Asymptomatic	31	(16%)	9	(13%)	9	(6%)	1	(2%)

Supplementary Table 2: Severity by age

	Severity		
Age groups	Severe	Mild	Asymptomatic
≤15	0 (0%)	24 (16%)	18 (58%)
15-60	3 (18%)	104 (69%)	12 (29%)
>60	10 (82%)	23 (15%)	1 (3%)
Total	13 (100%)	151 (100)	31 (100%)

Supplementary Table 3: Sera tested and positive by PRNT and ELISA stratified by days after onset of illness

Days after onset*	PRNT			Spike RBD ELISA	
	No tested	PRNT90 No (%) positive	PRNT50 No (%) positive	No tested	No (%) positive
0-7	35	11 (31%)	12 (34%)	9	2 (22%)
8-14	24	21 (88%)	23 (96%)	13	9 (69%)
15-21	15	15 (100%)	15 (100%)	14	14 (100%)
22-28	11	11 (100%)	11 (100%)	10	10 (100%)
29-60	93	91 (98%)	93 (100%)	84	84 (100%)
61-90	54	54 (100%)	54 (100%)	43	43 (100%)
91-150	35	34 (97%)	34 (97%)	16	32 (100%)
151-209	26	26 (100%)	26 (100%)	26	26 (100%)

* For asymptomatic individuals, days after first positive RT-PCR result.

Supplementary figure 1. Correlation between $PRNT_{90}$, and ELISA (left) and $PRNT_{50}$ and ELISA (right) in COVID-19 patients (n=231 pairs)

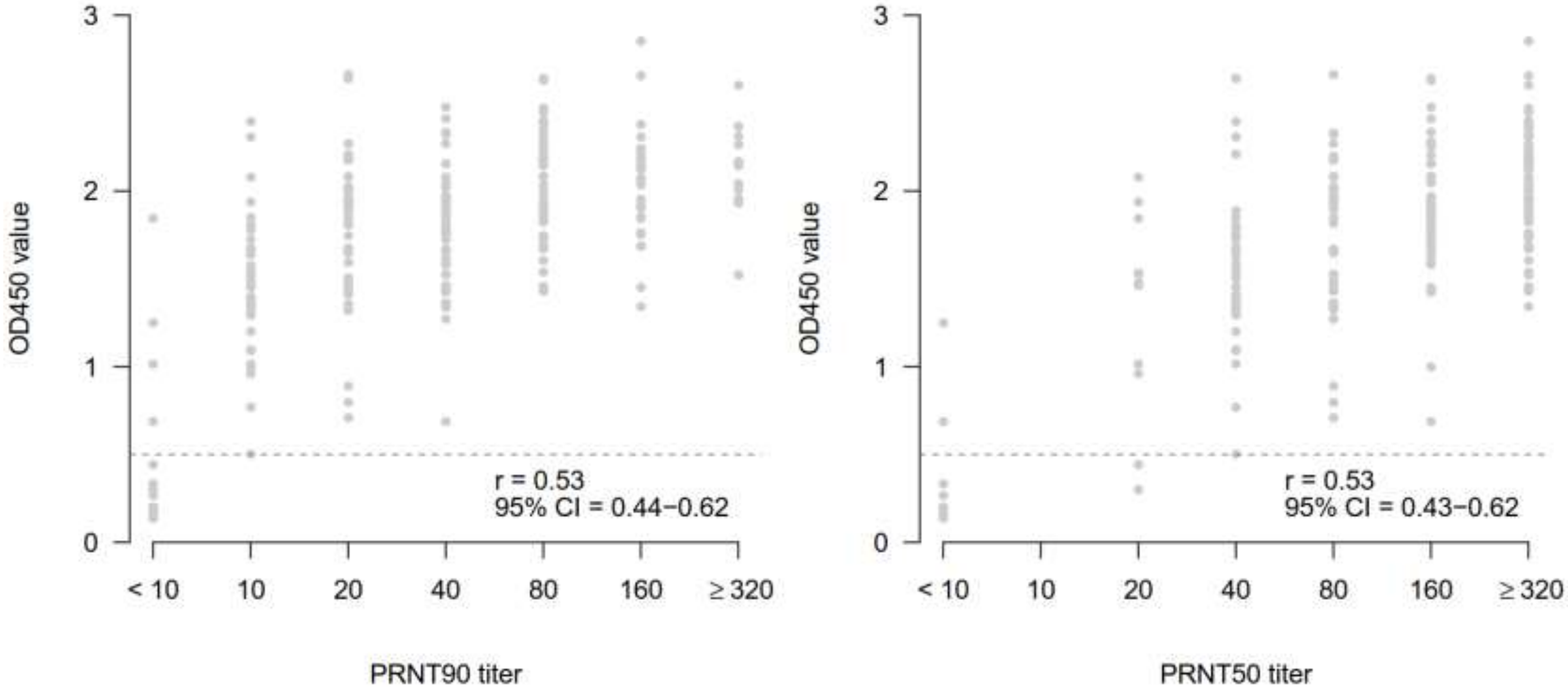

Supplement: Supplementary file 1 — Supplementary Information [file 41467_2020_20247_MOESM1_ESM.pdf]
