# Supplementary material for: Neutralizing antibody titres in SARS-CoV-2 infections
Source: Nat Commun. 2021 Jan 4;12:63. doi: 10.1038/s41467-020-20247-4 (PMC7782739; doi:10.1038/s41467-020-20247-4)
Supplement: Supplementary file 3 — Reporting Summary [file 41467_2020_20247_MOESM3_ESM.pdf]

nature research

Corresponding author(s): Malik Peiris

Last updated by author(s): 6 April 2020

Reporting Summary

Nature Research wishes to improve the reproducibility of the work that we publish. This form provides structure for consistency and transparency in reporting. For further information on Nature Research policies, see Authors & Referees and the Editorial Policy Checklist .

Statistics

For all statistical analyses, confirm that the following items are present in the figure legend, table legend, main text, or Methods section.

n/a	Confirmed	
☐	☒	The exact sample size (*n*) for each experimental group/condition, given as a discrete number and unit of measurement
☐	☒	A statement on whether measurements were taken from distinct samples or whether the same sample was measured repeatedly
☐	☒	The statistical test(s) used AND whether they are one- or two-sided<br>*Only common tests should be described solely by name; describe more complex techniques in the Methods section.*
☐	☒	A description of all covariates tested
☐	☒	A description of any assumptions or corrections, such as tests of normality and adjustment for multiple comparisons
☐	☒	A full description of the statistical parameters including central tendency (e.g. means) or other basic estimates (e.g. regression coefficient) AND variation (e.g. standard deviation) or associated estimates of uncertainty (e.g. confidence intervals)
☐	☒	For null hypothesis testing, the test statistic (e.g. *F*, *t*, *r*) with confidence intervals, effect sizes, degrees of freedom and *P* value noted<br>*Give P values as exact values whenever suitable.*
☒	☐	For Bayesian analysis, information on the choice of priors and Markov chain Monte Carlo settings
☒	☐	For hierarchical and complex designs, identification of the appropriate level for tests and full reporting of outcomes
☐	☒	Estimates of effect sizes (e.g. Cohen's *d*, Pearson's *r*), indicating how they were calculated

Our web collection on statistics for biologists contains articles on many of the points above.

Software and code

Policy information about availability of computer code

Data collection	No commercial or open source databases used.
Data analysis	The data analysis methods are all clearly described in the mansuscript.

For manuscripts utilizing custom algorithms or software that are central to the research but not yet described in published literature, software must be made available to editors/reviewers. We strongly encourage code deposition in a community repository (e.g. GitHub). See the Nature Research guidelines for submitting code & software for further information.

Data

Policy information about availability of data

All manuscripts must include a data availability statement. This statement should provide the following information, where applicable:

- Accession codes, unique identifiers, or web links for publicly available datasets
- A list of figures that have associated raw data
- A description of any restrictions on data availability

Data used in the analysis is provided as a source data file

Field-specific reporting

Please select the one below that is the best fit for your research. If you are not sure, read the appropriate sections before making your selection.

☒ Life sciences ☐ Behavioural & social sciences ☐ Ecological, evolutionary & environmental sciences

For a reference copy of the document with all sections, see nature.com/documents/nr-reporting-summary-flat.pdf

Life sciences study design

All studies must disclose on these points even when the disclosure is negative.

Sample size	The sample size was based on patient specimen availability and all the eligible clinical specimens that met the study inclusion criteria were included in the
Data exclusions	No data exclusions for the main outcome variable which was the neutralization titres. The ELISA assay results was a minor outcome and all available data was included.
Replication	All assays were done in duplicate, as usual for these types of studies.
Randomization	Not relevant. An observational study. No intervention investigated
Blinding	Not relevant. An observational study. No intervention investigated

Reporting for specific materials, systems and methods

We require information from authors about some types of materials, experimental systems and methods used in many studies. Here, indicate whether each material, system or method listed is relevant to your study. If you are not sure if a list item applies to your research, read the appropriate section before selecting a response.

Materials & experimental systems

n/a	Involved in the study	
☒	☐	Antibodies
☐	☒	Eukaryotic cell lines
☒	☐	Palaeontology
☒	☐	Animals and other organisms
☐	☒	Human research participants
☐	☒	Clinical data

Methods

n/a	Involved in the study	
☒	☐	ChIP-seq
☒	☐	Flow cytometry
☒	☐	MRI-based neuroimaging

Eukaryotic cell lines

Policy information about cell lines

Cell line source(s)	Vero-E6 cells (ATCC CRL-1586)
Authentication	Cell lines obtained from ATCC. Original cell stocks maintained in liquid N2 storage and each thawed aliquot discarded after 20 cell passages.
Mycoplasma contamination	Confirmed to be free of mycoplasma using two methods. A cell culture based kit from Invivogen. PlasmoTest™ - Mycoplasma Detection Kit and a PCR assay from ABM. https://www.abmgood.com/pcr-mycoplasma-detection-kit-g238.html
Commonly misidentified lines (See ICLAC register)	No commonly misidentified cell lines used.

Human research participants

Policy information about studies involving human research participants

Population characteristics	Patient recruiting criteria have been described. All eligible patients who consented have been included.
Recruitment	Patients who were discharged from hospital after SARS-CoV-2 infection were invited to participate. All those consenting were included
Ethics oversight	Institutional Review Board approval with oversight of each hospital from which patients were recruited was obtained and this is detailed in the manuscript.

Note that full information on the approval of the study protocol must also be provided in the manuscript.

Clinical data

Policy information about clinical studies

All manuscripts should comply with the ICMJE guidelines for publication of clinical research and a completed CONSORT checklist must be included with all submissions.

Clinical trial registration	This is not a clinical trial
Study protocol	An observational study on a patient cohort
Data collection	Patients were recruited and clinical data recorded from 21st January 2020 to 31st July 2020. Sera were tested by the relevant serological assays batch-wise subsequently.
Outcomes	Relevant outcomes were the SARS-CoV-2 neutralizing antibody titres and ELISA IgG antibody as quantified by optical density.
