# Supplementary material for: Neutralizing antibody titres in SARS-CoV-2 infections
Source: Nat Commun. 2021 Jan 4;12:63. doi: 10.1038/s41467-020-20247-4 (PMC7782739; doi:10.1038/s41467-020-20247-4)
Supplement: Supplementary file 2 — Peer Review File [file 41467_2020_20247_MOESM2_ESM.pdf]

Editorial Note: This manuscript has been previously reviewed at another journal that is not operating a transparent peer review scheme. The manuscript was considered suitable for publication without further review at *Nature Communications.*
